# The Risk of Metabolic Syndrome in Patients with Rheumatoid Arthritis: A Meta-Analysis of Observational Studies

**DOI:** 10.1371/journal.pone.0078151

**Published:** 2013-10-25

**Authors:** Jianming Zhang, Lingyu Fu, Jingpu Shi, Xin Chen, Yongze Li, Bing Ma, Yao Zhang

**Affiliations:** 1 Department of Clinical Epidemiology and Evidence-based Medicine, the First Affiliated Hospital, China Medical University, Shenyang, China; 2 Key Laboratory of AIDS Immunology of Ministry of Health, Department of Laboratory Medicine, No 3 Hospital of China Medical University, Shenyang, Liaoning, China; University of Birmingham, United Kingdom

## Abstract

**Background:**

Observational studies suggest an association between the incidence of rheumatoid arthritis (RA) and the prevalence of metabolic syndrome (MetS). However, the relationship between RA and MetS is controversial and research in this area is currently lacking.

**Objective:**

The aim of this study was to assess whether the prevalence of MetS was higher in a group of RA patients compared to subjects without RA.

**Design:**

A PubMed database search was conducted during April 2013 to identify observational studies of RA and risk of MetS. Reference lists of retrieved articles were also reviewed. Two authors independently extracted information on the study design, the characteristics of the study participants, exposure and outcome assessments, and the method used to control for potential confounding factors. A random-effects model was used for the risk estimates.

**Results:**

Our meta-analysis of four cross-sectional controlled studies plus eight case-control studies involving a total of 2283 cases and 4403 controls identified a significant association between RA and risk of MetS, with an overall OR of 1.24 (95% CI, 1.03-1.50).

**Conclusion:**

This meta-analysis provides further evidence supporting patients with RA have a higher prevalence of MetS than subjects without RA. In addition, the geographic region of the population and the criteria used for MetS diagnosis could influence the association. However, these observations would need to be evaluated using prospective, randomized studies.

## Introduction

Metabolic syndrome (MetS), also known as syndrome X and insulin resistance syndrome, was first described by Reaven in 1988 [1]. It is a cluster of classical cardiovascular (CV) risk factors (obesity, glucose intolerance, dyslipidaemia, and hypertension), the combination of which is thought to be associated with CV risk beyond the sum of the individual components [2]. MetS is responsible for a three-fold increase in the risk of atherosclerotic cardiovascular diseases（CVDs） and also increases mortality from CVD, as well as all-causes, in the general population [3]. Recent years have seen an accumulation in the number of patients with MetS, especially in developed countries. Several studies have demonstrated that inflammatory processes are involved in the pathogenesis of the MetS and there is evidence that suggests that components of CV risk increase the inflammatory burden in rheumatoid arthritis (RA). RA is a systemic inflammatory disorder characterized by chronic symmetric and erosive synovitis that preferentially affects peripheral joints, with a prevalence of 0.5–1% in the population[4]. Emerging epidemiological evidence suggests that CVDs account for approximately 50% of all RA-associated deaths [5]. Previous evidence exists that shows an association between RA and MetS, but their results on the prevalence of the syndrome in RA patients were conflicting [6]. Given the inconsistency of the existing literature and the insufficient statistical power of primary studies, we conducted a meta-analysis to investigate the relationship between RA and MetS, whereby we calculated the magnitude of the association across all studies.

## Methods

### Search strategy

Our analysis was reported in accordance with the Meta-analysis of Observational Studies in Epidemiology guidelines for meta-analysis of observational studies [7]. We conducted MEDLINE and EMBASE database searches during April 2013 in order to identify articles that reported the prevalence of MetS in RA patients. We used the key word "metabolic syndrome" in combination with "rheumatoid arthritis" with no restrictions. We then examined the reference lists of the obtained articles. In addition, we reviewed the reference lists of retrieved papers and recent reviews. 

### Study selection

Two reviewers independently screened the abstracts and titles of the search results. A second screening was based on full-text review. Studies were included in our meta-analysis if they met the following criteria: 1) the study design was observational; 2) the patients included in the study were RA patients, all of whom fulfilled the American College of Rheumatology (ACR) classification criteria for RA; 3) the outcome of interest was the prevalence of MetS in RA patients; 4) odds ratios (OR) and corresponding 95% confidence intervals (CIs) (or data to calculate them) were reported. Only studies where age and sex were similar between the two patient groups were included in the meta-analysis.

### Data extraction and quality assessment

We extracted all data using a standardized data collection form. General characteristics of the study (first author; year of publication; country; characteristics of study population, including age, sex and race at baseline; number of RA; study design; value of the Disease Activity Score using 28 joints (DAS28); duration of disease; the criteria of RA; the assessment of MetS and the number of MetS, score of the study) were recorded, where available, and double-checked. Two reviewers independently conducted the literature search, study selection and data extraction. Studies were evaluated for eligibility on the basis of predefined, set criteria. Disagreements between the two reviewers were resolved by discussion.

### Statistical analysis

Homogeneity test was performed with the use of *Q* statistic at the *P*<0.10 level of significance. We also calculated the *I* ^2^ statistic, a quantitative measure of inconsistency across studies [8]. A random-effects model was used to calculate the combined OR. Results from the random-effects model, which considers both within- and between-study variation [9], were used to compute the summary risk estimate. Subgroup analyses according to geographic region and differing evaluation standards of MetS were performed to assess the potential modifying effect of these variables on outcomes. We also conducted a sensitivity analysis to investigate the influence of a single study on the overall risk estimate, by omitting one study at a time. Heterogeneity between the results of different studies was examined using *χ* ^2^ tests (*P*<0.10 was considered to be statistically significant) and *I* ^2^ tests (*I* ^2^>50%: significant heterogeneity; *I* ^2^<25%: insignificant heterogeneity) [8]. To estimate the extent of publication bias, Begg's correlation and Egger's regression for publication bias were used [10,11]. Visual inspection of publication bias using a funnel plot of the data was not employed because bias may be incorrectly inferred if studies are heterogeneous [12]. All analyses were performed using STATA version 11.0 (Stata, College Station, TX, USA). *P*<0.05 was considered statistically significant, except where otherwise specified. 

## Results

### Selected studies and characteristics

**Figure 1:**
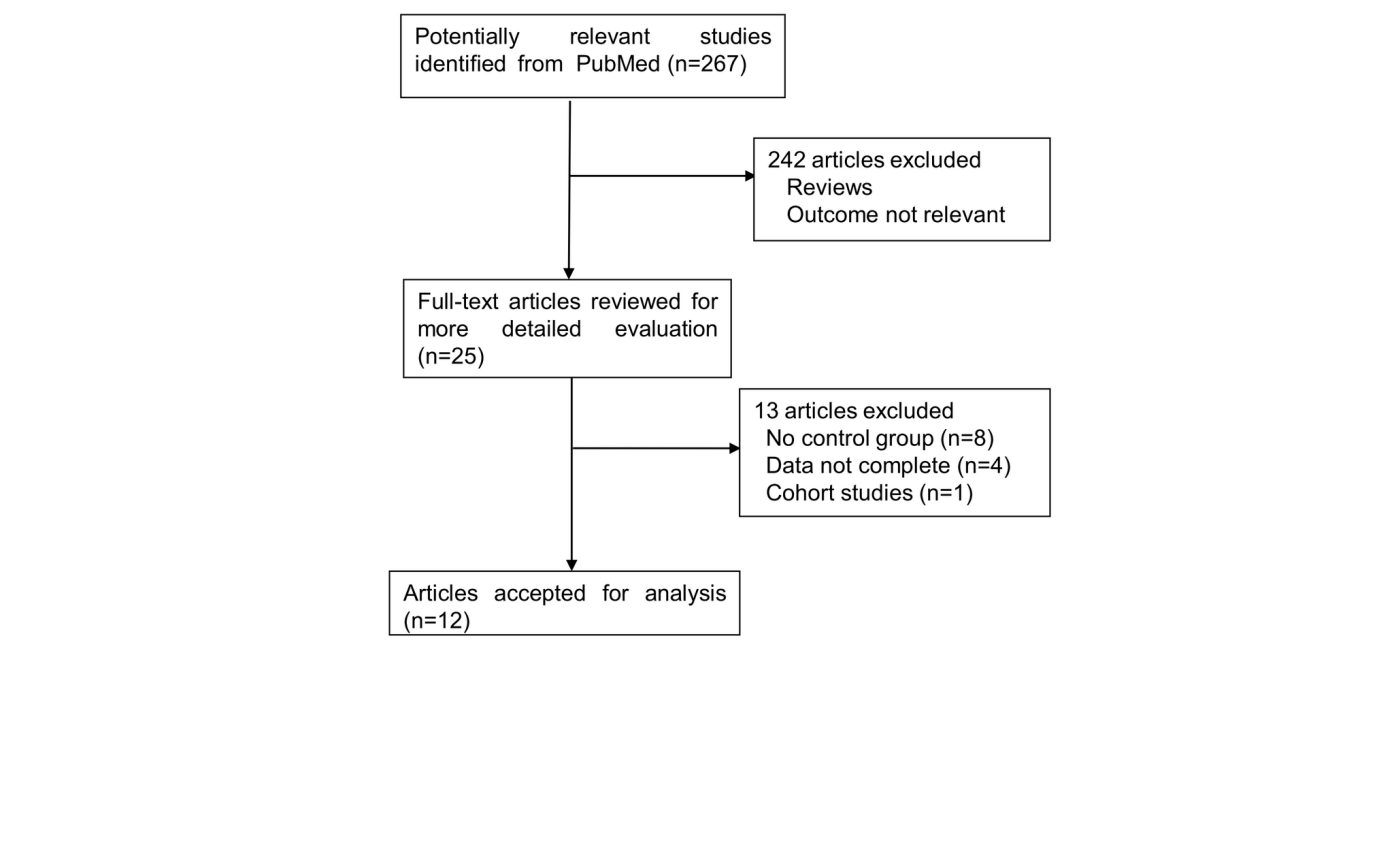
Study flow chart.

Of the 267 reports initially identified (including electronic and manually retrieved), we excluded 242 studies that did not investigate the association between MetS and RA after the first screening focusing on abstracts and titles. Of the 25 articles selected for detailed evaluation, the data of one study was deemed inconsistent [13], eight studies did not have a control group [5,14-20] and one study used a cohort study design [21]. Another three studies were excluded for having incomplete data [22-24]. Twelve studies remained for inclusion in our meta-analysis [25-36]. A flow chart showing the study selection process is presented in Figure **1**. The characteristics of the 12 studies are presented in Table **1**. These studies were published between 2006 and 2013. Of these, three studies were conducted in North America, one in South America, three in Europe, and five in Asia. Sample size ranges from 43 to 1398 participants, with a total of 2283 cases and 4403 controls. RA patients fulfilled the diagnostic criteria of the American College of Rheumatology (ACR). Disease activity was evaluated using DAS28. 

**Table 1:** Characteristics of the studies included in the meta-analysis.

Study	Country	Duration (year)	Number RA/CT (female)	Age(year) RA/CT	DAS28	Research type	Ascertainment of RA	Race RA/CT	NCEP RA/CT	WHO RA/CT	IDF RA/CT	Score
Santos et al., 2010	Portugal	7.6	98/102 98/102(F)	49.2±13.7/ 47.7±13.4	4.24	Case-control	ACR	Caucasian 93%	25/16	NA	NA	8
Dao et al., 2010	Viet Nam	1.75	105/105 105/105(F)	56.3/55.7	4.1	Cross-sectional controlled	ACR1987	Asian 100%	34/19	20/13	43/24	7
Crowson et al., 2011	USA	7.0	232/1241 174/681(F)	58.8±12.8/ 63.9±9.2	NA	Cross-sectional controlled	ACR1987	Caucasian92%	76/316	NA	NA	7
Karvounaris et al., 2006	Greece	9.52	200/400 147/294(F)	63±11/ 63±11	5.1	Cross-sectional controlled	ACR	Caucasian100%	88/164	NA	NA	7
Cunha et al., 2012	Brazil	10.86	283/226 233/192(F)	56.8±12.3/ 44.5±8.3	3.32	Case-control	ACR	Caucasian 86%	111/44	NA	NA	8
MOK et al., 2011	Hong Kong	5.3	699/1398 566/1132(F)	53.3±12.0/ 52.9±12.0	NA	Case-control	ACR1987	Asian 100%	137/278	NA	NA	7
Chung et al., 2008	USA	5.5	154/85 104/55(F)	54/52	3.1	Case-control	ACR	Caucasian 88%	54/19	55/8	NA	9
KARIMI et al., 2011	Iran	8	92/96 92/96(F)	48.3±14.6/ 42.2±9.9	4.3	Case-control	ACR	Asian 100%	25/34	18/21	NA	7
Karakoc et al., 2012	Turkey	7.6	54/52 47/43(F)	49.76±11.15/ 47.05±9.75	3.79	Case-control	ACR	Caucasian100%	NA	NA	23/5	6
Sahebari et al., 2011	Iran	5.5	120/500 106/431(F)	45.5±13/ 45.6±12	4.5	Case-control	ACR1987	Asian 100%	54/269	NA	37/171	6
Ormseth et al., 2013	USA	NA	162/89 144/56(F)	54/53	NA	Case-control	ACR1987	Caucasian86%	58/18	NA	NA	7
Lee et al., 2013	Korea	3.5	84/109 84/109(F)	50.6±11.3/ 48.3±11.3	3.49	Cross-sectional controlled	ACR1987	Asian 100%	16/17	NA	NA	9

### Main analysis

**Figure 2:**
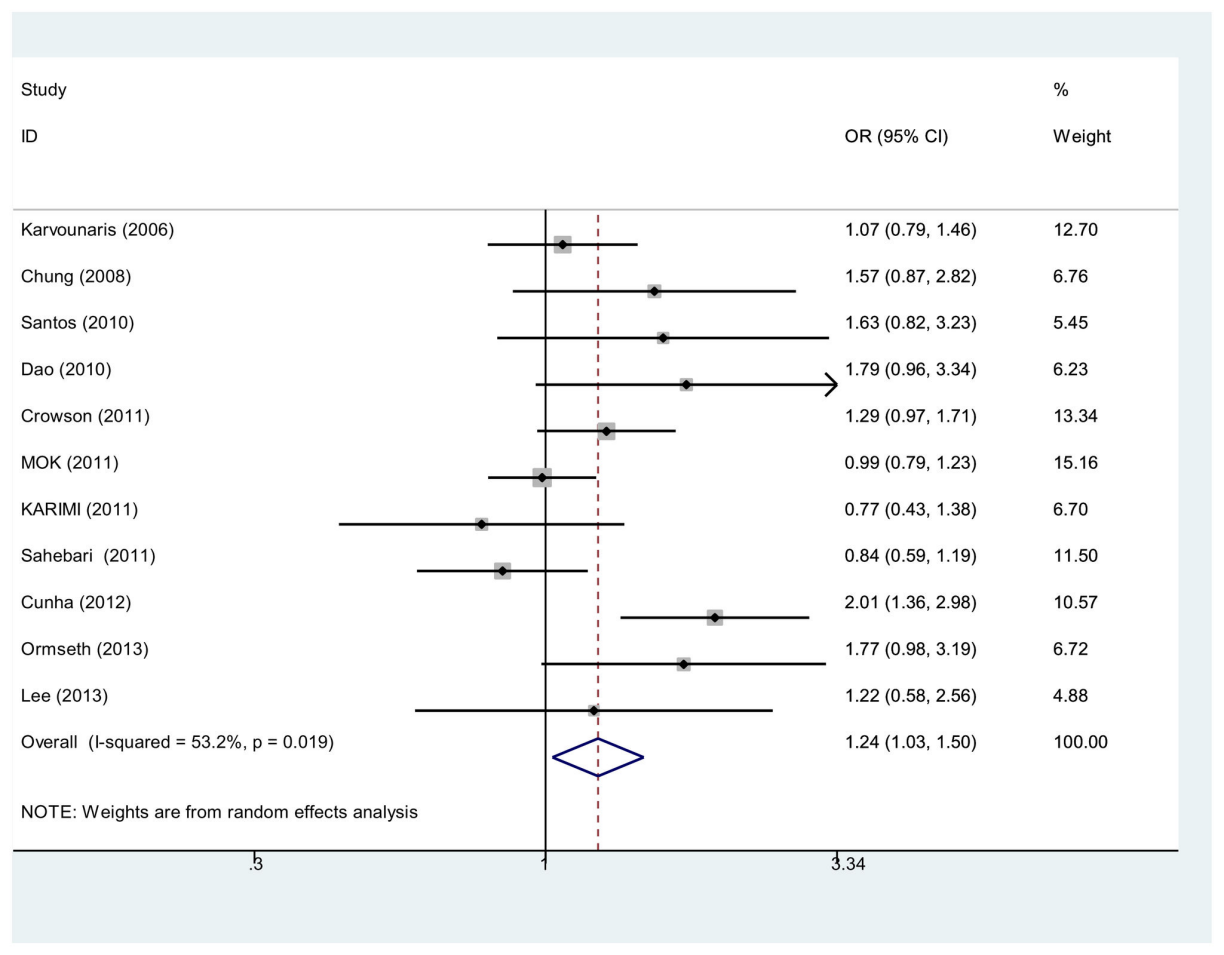
Odds ratios and 95 % conﬁdence intervals (CIs) of individual studies and of pooled data for the association between Metabolic Syndrome and Rheumatoid Arthritis in all subjects.

MetS patients in the included studies were all confirmed with NCEP-ATP III. Odds ratios (OR) were used to evaluate the association between RA and MetS. Substantial heterogeneity was observed across studies (*P*_heterogeneity_ = 0.019, *I* ^2^ = 53.2%), therefore a random-effects model was used. The overall OR in our study was 1.24 (95% CI, 1.03-1.50) (Figure **2**)**.**

### Meta-regression and subgroup meta-analysis

**Figure 3:**
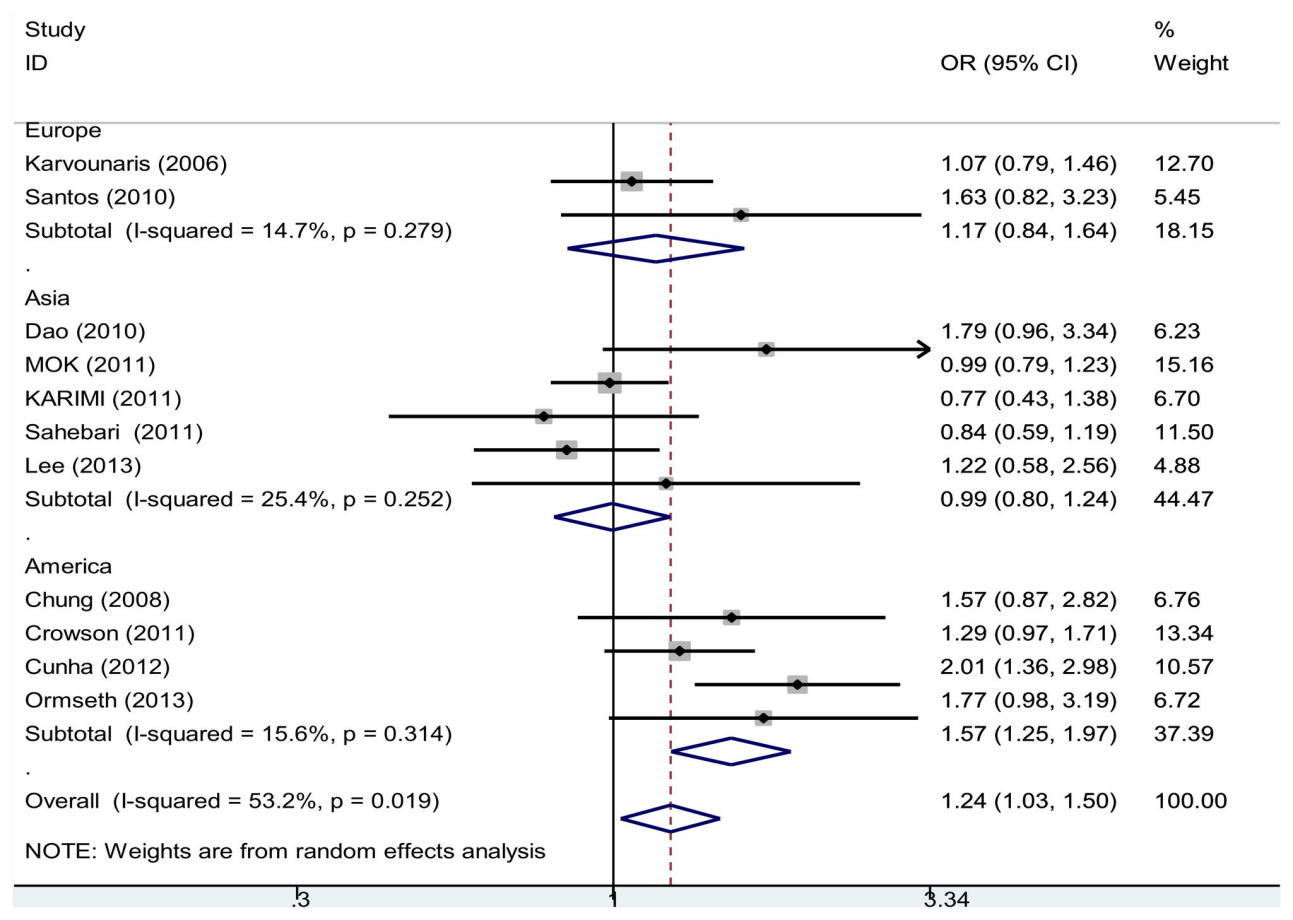
Odds ratios and 95 % conﬁdence intervals (CIs) of individual studies and of pooled data for the subgroup analysis by the geographic region.

To explore the source of heterogeneity, four variables were tested in meta-regression analysis. “Geographic region (Asia, Europe and America)” was significantly related to high heterogeneity. Figure **3** shows the results of subgroup analysis with reference to geographic region. Pooled analysis was conducted for the American, Asian, and European subgroups in order to explore the relationship between RA patients and the risk of MetS in different areas. The combined ORs between RA and MetS of the European and Asian populations were 1.17 (95% CI, 0.84-1.64) and 0.99 (95% CI, 0.80-1.24), respectively. There was no evidence of heterogeneity in the European subgroup (*P*_heterogeneity_=0.279, *I* ^2^=14.7%) or the Asian subgroup (*P*_heterogeneity_=0.252, *I* ^2^=25.4%). However, there was a more pronounced association in the American subgroup, OR=1.57 (95% CI, 1.25-1.97). Heterogeneity was also not observed in this subgroup population (*P*_heterogeneity_=0.314, *I* ^2^=15.6%). 

**Figure 4:**
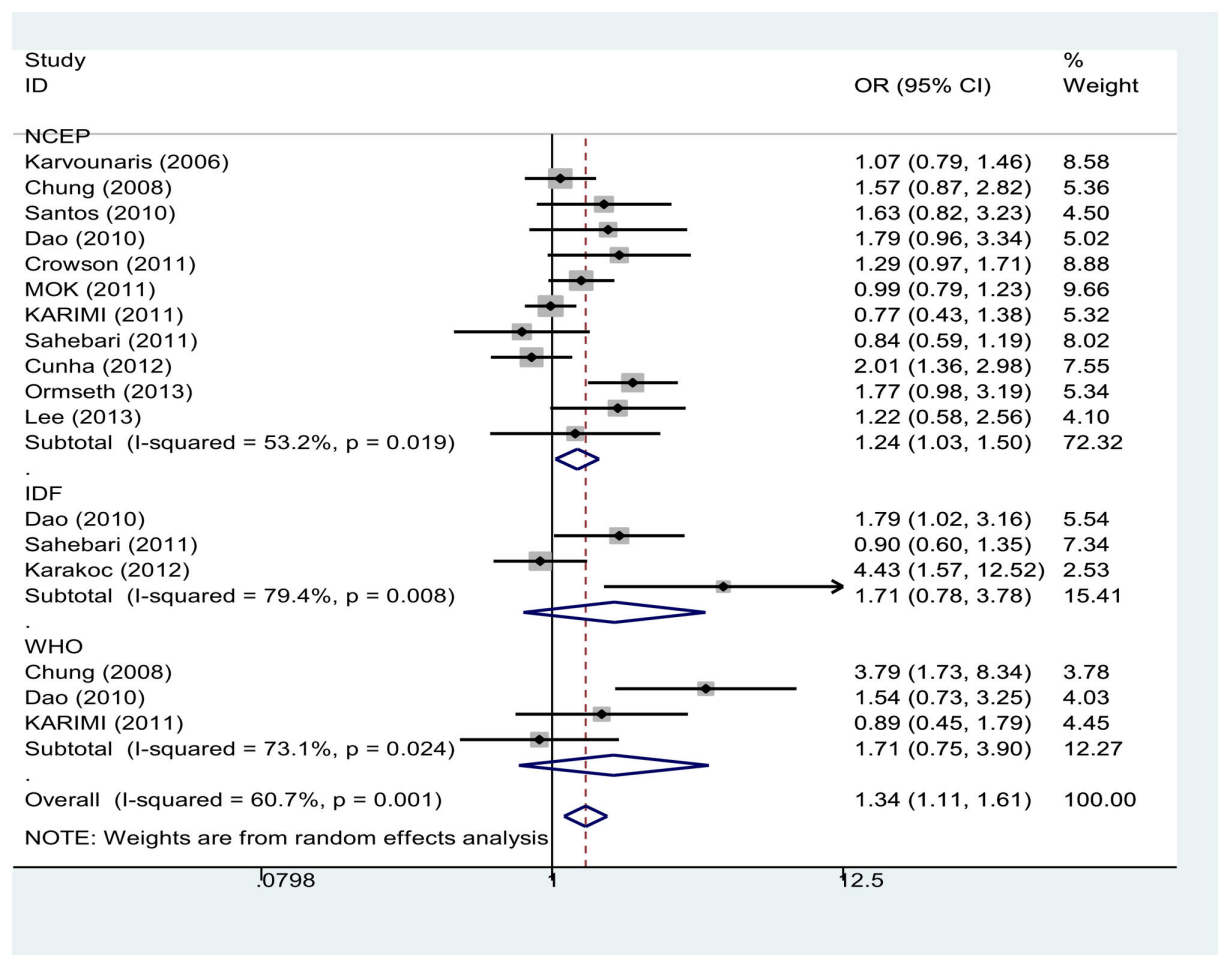
Odds ratios and 95 % conﬁdence intervals (CIs) of individual studies and of pooled data for the affect factor by different evaluate standard of Metabolic Syndrome.

In order to explore the influence of the differing evaluation standards of MetS, the studies were separated into NCEP-ATP III, WHO and IDF subgroups according to the evaluation standard used to diagnose MetS. The combined overall OR between RA and MetS was 1.34 (95% CI, 1.11-1.61). We identified an association between RA and MetS in the NCEP-ATP III subgroup (OR=1.24 (95% CI, 1.03-1.50)). No association was identified between RA and MetS in either the WHO subgroup (OR=1.71 (95% CI, 0.75-3.90) or the IDF subgroup (OR=1.71 (95% CI, 0.78-3.78) (Figure **4**).

### Sensitivity analysis and publication bias

To test the robustness of our findings, sensitivity analysis was conducted. The analysis investigated the influence of a single study on the overall risk estimate by omitting one study at a time, yielding a narrow range of ORs; 1.28 (95% CI, 1.01–1.64) to 1.56 (95% CI, 1.15–2.11). In addition, no single study substantially contributed to the heterogeneity observed across all studies. No evidence of publication bias was found with regards to RA patients in relation to MetS risk, as indicated by Begg rank correlation test (*P*=0.392) and Egger linear regression test (*P*=0.157). 

## Discussion

This meta-analysis of 12 observational studies involving 2283 cases and 4403 controls supports a significant association between RA and MetS. The presence of MetS in patients could pose 1.24 times higher risk for RA. 

MetS, a set of CV risk factors, has been assessed aiming at predicting the risk for CVD [28]. Despite questions about the existence of MetS, it is undoubtedly paramount to identify the presence of its components and establish adequate management strategies so that diabetes- and CVD-associated morbidity and mortality can be reduced [37]. Currently, CVD is the major cause of death in patients with RA, and acute myocardial infarction can be up to four times more frequent in these patients [28,38]. Given the increased prevalence of CVD in RA patients, it would not be surprising to see an increased prevalence of MetS in these patients. MetS is also considered an inflammatory state [39]. There is increasing evidence that insulin resistance, the basic metabolic disturbance of MetS, is not only associated with the abundance of inflammatory cytokines but also is a direct result of this burden [40,41]. It is directly correlated with levels of interleukin 6, tumor necrosis factorα (TNFa), C-reactive protein (CRP), and erythrocyte sedimentation rate (ESR) or coronary calcification [42-45]. The adipose tissue may play a key role in the relationship between RA of MetS. It is independently associated with each of the other individual MetS components and is generally regarded as a prerequisite risk factor for the diagnosis of this condition. Areas of active investigation focus on the molecular bases of metabolic inflammation and potential pathogenic roles in insulin resistance and CVD. An increased accumulation of macrophages occurring in obese adipose tissue has emerged as a key process in metabolic inflammation [46]. Besides the central role of lipid storage, it has a major endocrine function secreting several hormones [47]. Adipokines exert potent modulatory actions on target tissues and cells involved in rheumatic disease, including cartilage, synovium, bone, and various immune cells [48]. Therefore, it had been hypothesized that MetS may be the risk factor of RA patients. Through the subgroup analysis, we found that the association between RA and MetS was significant in the American subgroup. However, no relationship was found in either the Asian or the European subgroups, which prompted us to conclude that the American subgroup was the susceptible population and that the presence of MetS was the risk factor in the development of RA. This may be due to the lifestyle and dietary pattern in different areas and their differing immune statuses. American dietary pattern are high in both fat and calories. This may increase their likelihood of developing MetS. There is evidence that under a given body mass index (BMI), body fat percentage is greater in Caucasians than Asians [49]. Therefore, American MetS patients may be predisposed to greater immune risk; however, there is no available information in the literature regarding MetS in this population. 

To date, six definitions for MetS have been established: the Joint Consensus 2009 of the International Diabetes Federation (IDF) Task Force, National Heart, Lung, and Blood Institute, American Heart Association, World Heart Federation, International Atherosclerosis Society and International Association for the Study of Obesity [50], the IDF 2005 [51], the National Cholesterol Education Program (NCEP) 2004 [52] and 2001 [53], The European Group for Study of Insulin Resistance (EGIR) 1999 [54], and the World Health Organization (WHO) 1998 [55]. Among them, those from the WHO, NCEP-Adult Treatment Panel III (NCEP-ATP III) and IDF are the most important for clinical use and therefore these were included in our subgroup analysis according to the diagnosis of MetS. These definitions have many similarities but differ in the standard of glucose, HDL-c, triglycerides, obesity and hypertension. Our analysis showed that the same people using different standards for diagnosing MetS may give different results. Hence, people in different regions should select appropriate standards for diagnosing MetS. It is important to note that the number of participants in each subgroup differs and this may have an effect on our analysis results.

Meta-analyses are categorized as observational studies as opposed to tentative research. Therefore, they are subject to bias [56]. There are several issues that arose from this systematic review. Firstly, among the 12 studies, there was no prospective study with high ability of etiology inference included. The collected information in cross-sectional studies can only reflect the individual disease condition and cannot establish causal association. Case-control studies are inevitably affected by a variety of bias influences, especially selective bias, and therefore their results are not as good as those of a prospective cohort study. Secondly, substantial heterogeneity was observed among studies, which was not surprising given the differences in characteristics of populations and adjustment for confounding factors. We explored the issue of heterogeneity via subgroup analysis. The results showed that geographic region contributed to the source of heterogeneity. However, each subgroup analysis only analyses from an angle. If the sources of the heterogeneity are from much aspect, it is unable to be solved by the present method. Thirdly, information on menopausal status and hormone replacement therapy use was not available for the present analysis. As the participants in four of the studies were all female, this may affect our results. Fourthly, according to our study, MetS has been associated with an increased prevalence of RA, but the role of different characteristics of the disease, such as disease duration and activity , and the frequency of treatment with methotrexate (MTX) and glucocorticoids are not well defined. Several components of MetS are directly influenced by RA disease activity and its treatment, because all the collected information from the cross-sectional study and case-control study, almost all the patients including in our study have accepted the corticosteroids treatment for a few time. Corticosteroids may improve insulin sensitivity in patients with RA [57], Other studies have shown that a high disease activity is correlated with adverse lipid profile [58,59] and insulin resistance [45]. Concurrently, endogenous glucocorticoid hormones regulate high density lipoprotein (HDL) concentration in plasma by increasing synthesis and secretion of HDL by the liver [60]. Therefore, the use of these drugs in patients with RA may decrease the prevalence of MetS. MTX is the common treatment for patients with RA. It may result in a reduction in CVD-related mortality as well as improvements in lipid and glucose profiles, such as lower triglyceride (TG) levels, higher HDL-C levels and lower plasma glucose [16]. These indicators could affect the incidence of MetS. Fifthly, for certain meta-analyses, relatively fewer studies were included, which lowered their ability to better estimate the effects. However, all studies in our analysis are relatively new and all of them were published post-2006. Finally, any meta-analysis has inherent limitations due to it being based on the summary of previous published sources and is limited by many factors. Therefore, cautious interpretation of results is recommended. Our study shows a significantly increased prevalence of MetS in RA patients compared to non-RA subjects. Recognition of MetS in RA patients who have not yet developed CVD could provide a valuable opportunity for preventative intervention. However, the statistical evaluation and clinical evaluation of our results are not the same. Due to the limitations of our meta-analysis, more research is needed to understand the reasons for these metabolic changes in RA and the impact of MetS on development of CVD in RA patients, especially prospective studies including multiple races.

## Conclusion

Our meta-analysis indicates that MetS may be the major contributing risk factor for the development of RA. In addition, geographic region and the criteria used for RA diagnosis could influence any association. However, further prospective randomized studies are necessary to evaluate these observations. 

## Supporting Information

Checklist S1 **PRISMA Checklist of this meta-analysis.**
(DOC)
